# Role of vaccination in economic growth

**DOI:** 10.3402/jmahp.v3.27044

**Published:** 2015-08-12

**Authors:** Sibilia Quilici, Richard Smith, Carlo Signorelli

**Affiliations:** 1 Sanofi Pasteur MSD, Lyon, France; 2 Faculty of Public Health and Policy, London School of Hygiene & Tropical Medicine, London, UK; 3 Department of Biomedical, Biotechnological & Translational Sciences (S.Bi.Bi.T), Public Health Unit, University of Parma, Parma, Italy

**Keywords:** Vaccination, macroeconomic, productivity, economic growth, investment

## Abstract

The health of a population is important from a public health and economic perspective as healthy individuals contribute to economic growth. Vaccination has the potential to contribute substantially to improving population health and thereby economic growth. Childhood vaccination programmes in Europe can offer protection against 15 important infectious diseases, thus preventing child fatalities and any serious temporary and permanent sequelae that can occur. Healthy children are more able to participate in education, thus preparing them to become healthy and productive adults. Vaccination programmes can also prevent infectious diseases in adolescents, thus allowing them to continue their development towards a healthy adulthood. Protecting adults against infectious diseases ensures that they can fully contribute to productivity and economic development by avoiding sick leave and lower productivity. Vaccination in older adults will contribute to the promotion of healthy ageing, enabling them to assist their familiy with, for instance, childcare, and also help them avoid functional decline and the related impacts on health and welfare expenditure. Effective vaccination programmes for all ages in Europe will thus contribute to the European Union's 2020 health and economic strategies. Indeed, beyond their impact on healthcare resources and productivity, reductions in mortality and morbidity also contribute to increased consumption and gross domestic product. Therefore, assessment of the value of vaccines and vaccination needs to consider not just the direct impact on health and healthcare but also the wider impact on economic growth, which requires a macroeconomic analysis of vaccination programmes.

The European financial and economic crisis, which started in 2008, has put pressure on healthcare budgets, resulting in cuts that are often short term and leading to unprecedented consequences on healthcare systems and the health of European citizens (1). In times of economic crisis and austerity, investments in health may need to be frozen. However, since health is closely linked to productivity and therefore the economic viability of individuals, populations, and nations, this may not be the wisest long-term strategy. Good health drives higher incomes through a number of mechanisms: education, labour productivity, tax contributions, investment, and savings (2–4) (Fig. 1). A recent European study estimated that the return on investment for each dollar of government spending in health was $4 (5). It has been shown that population health can operate through multiple channels, contributing to economic growth, which can in turn generate additional resources to reinvest into health, generating a virtuous cycle. Healthy children tend to achieve better educationally and to have better cognitive function, healthy adults tend to work longer and more productively, and healthy populations tend to save more and to attract more foreign investment contributing to capital accumulation, job creation, and technological progress (6). It is generally thought, especially in developing countries, that higher incomes promote better health through improved nutrition, sanitation, adoption of healthy lifestyles, and the ability to purchase better, high-quality healthcare.

**Fig. 1:**
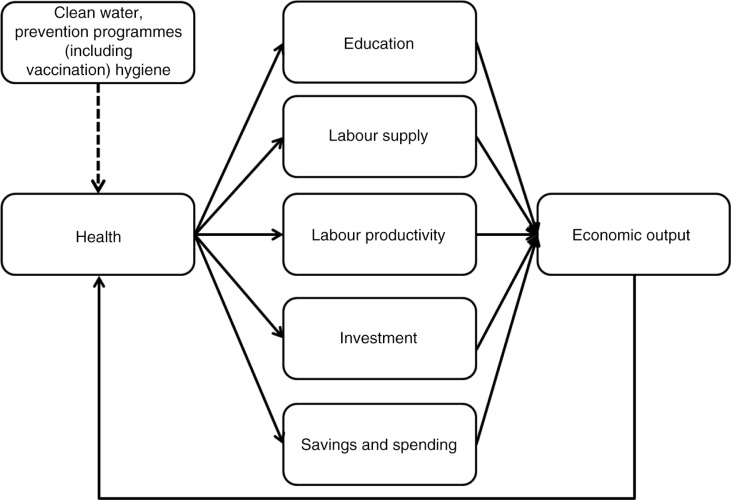
Potential mechanism for the link between health and economic output and the roles of clean water, prevention programmes, including vaccination, and hygiene (2–4).

Health enhancement is largely dependent on prevention, which can be considered to be the first level of healthcare (2). The importance of prevention was recognised many centuries ago by Desiderius Erasmus with his famous quote ‘Prevention is better than cure’. The primary objective of prevention is to protect health by avoiding diseases (primary prevention) and stopping or slowing the progress of diseases in their earliest stages (secondary prevention). As such, vaccination is recognised by many, including the World Health Organization, as one of the most cost-effective (and often cost-saving) primary prevention measures to help protect individuals and to promote public health.

Prevention programmes, such as vaccination, are often considered to have only return in the long run; thus, in situations where governments are looking to cut expenditure, they will look for short-term return and stop investing in prevention programmes. However, health should not be seen as an expenditure, or consumption good, but as an investment (7). In addition to its effectiveness in reducing disease and mortality, the benefits of vaccination have usually been measured in terms of the averted costs of medical care (8, 9). Sometimes consideration is also made of the immediate productivity loss to patients (as a result of illness or death) and their carers (10). However, in the longer term, it has been suggested that vaccines can increase lifetime productivity due to improved physical capacity, cognition, and educational outcomes through increased school attendance (11). Reductions in mortality and morbidity also contribute to increased consumption and gross domestic product (GDP) (6). For example, preliminary research suggested that a 5-year improvement in life expectancy can translate into 0.3 to 0.5% increased annual growth (6).

A framework to present these broader benefits has already been proposed, using the example of haemophilus influenza type b (Hib) vaccination (Table 2) and other available vaccines (6, 12). In this article, we aim to review the role of lifelong vaccination in economic growth, especially in Europe, and the need for a broader macroeconomic assessment framework for economic evaluations of vaccines.

## Paediatric vaccination programmes represent a long-term investment in future generations with a positive net present value for the society and the economy

Infant mortality rates (i.e., death of infants <1 year of age) reflect a country's economic and social conditions as well as the performance of its healthcare system. All European countries have achieved reductions in infant mortality rates since 1970, from an average of 25 deaths per 1,000 live births, to the current average of 4.2 per 1,000 births, corresponding to a cumulative reduction of over 80% (13). The reasons for this progress include improvements in sanitation, access to vaccination against infectious diseases, other public health measures, and wider social factors.

Investing in children's health is an investment in tomorrow's society. Good health from prenatal life to adolescence is a resource for social and economic development. The burden of ill health and impaired development in children has a multitude of effects. Sick children make additional demands on their parents, which can have an impact on the family's earning potential and can have detrimental consequences for their siblings. For example, the results from a study in South Africa have shown a significant association between coverage of measles vaccination and the level of school-grade attainment in siblings, suggesting that 1 year of schooling could be gained for every six children vaccinated against measles (6). Sick children also represent a cost for health and welfare systems that can sometimes continue into adult life. Poor cognitive and social development for children can create a lifetime of disadvantage, the legacy of which is often passed onto future generations.

From birth, a child is at risk of developing many severe infectious diseases that, in the absence of effective vaccines, can have serious consequences on their survival likelihood, as well as their physical and cognitive development (Table 1) (14). In the European Union (EU), childhood vaccination programmes protect against up to 15 different infectious diseases that could potentially result in high impairment with a huge impact on future human capital development (15). These diseases are tuberculosis, rotavirus, diphtheria, tetanus, pertussis, poliomyelitis, Hib, hepatitis b, pneumococcal disease, meningococcal disease, measles, mumps, rubella, varicella, and influenza. The benefits of infant vaccination against many of these diseases are often not taken into account, from a social and macroeconomic viewpoint. However, in the absence of these vaccines, substantial numbers of children would die, and many of those who would survive could develop mental and physical disabilities, preventing them from getting the most out of the education system and thereby impacting their productivity capacity when they reach adulthood (4). For example, up to 10% of Hib cases in children aged 2 months to 5 years are fatal, and up to 35% of the survivors can suffer from long-term permanent neurological sequelae such as deafness, blindness, mental retardation, epilepsy, and paralysis, which can affect their ability to go to school and to learn, which is in turn related to lower labour productivity and lifetime earnings that may result in less tax revenues and thus fewer resources available for public investments (Table 2) (12, 16).

**Table 1:** Potential medical impact of some vaccine-preventable infectious diseases (based on fact sheets available on the ECDC website) (14)

			Risk of		
			
Vaccine-preventable diseases	Age and population at risk of infection	Potential complications and medical impact	lifelong cognitive impairment	lifelong physical impairment	death
Measles	Can be contracted at any age	Pneumonia, encephalitis, death	x	x	x
Chickenpox	90% of cases in children aged <10 years. Fewer than 15% of chickenpox cases in people aged >15 years; most severe cases in adults, with chances of complications increasing with age	Encephalitis, secondary infections (severe streptococcus, skin infection), hepatitis, pneumonia: can be fatal in around 10% of cases	x	x	x
Pneumococcal disease	Any age but most likely to happen in children aged <2 years and adults aged >65 years	Bacterial meningitis, pneumonia, blood infection, septicaemia	x	x	x
Seasonal flu	Can be contracted at any age	Ear and sinus infections, pneumonia, heart inflammation, and death		x	x
Rotavirus gastroenteritis	Mostly in children aged <5 years	Severe dehydration (loss of 10% of weight in children), sometimes death			x
Whooping cough (pertussis)	Can be contracted at any age – most severe cases in babies <6 months of age	Coughing spells so bad that it is hard to eat, drink, or breathe. Can last for weeks and lead to pneumonia, seizures (jerking and staring spells), brain damage, or death	x	x	x
Hepatitis B	Chronic infection is most likely to develop in young babies. Most infections occur in adults in high-risk groups	Chronic infections can lead to inflammation of the liver, liver damage (called cirrhosis), and cancer		x	x
*Haemophilus influenza* type b (Hib)	Aged 2 months–5 years	Most common cause of bacterial meningitis in children before the introduction of the vaccination, leading to brain damage or death (up to 10% of cases)	x	x	x
Tetanus	The highest tetanus risk in Europe is found in the unvaccinated elderly	Painful tightening of muscles can lead to spasm, and death in 10% of cases		x	x
Polio	Can be contracted at any age	In children aged <5 years: paralysis of one leg is most common In adults: extensive paralysis of the chest and abdomen is more likely May lead to death		x	x
Diphtheria	Can be contracted at any age	Can lead to breathing problems, paralysis, heart failure, and even death		x	x
Meningococcal disease	Most frequently occurs in young children, but a second disease peak is observed among adolescents and young adults	Even when the disease is diagnosed early and adequate treatment is started, 5 to 10% of patients die, typically within 24 to 48 hours after the onset of symptoms. Bacterial meningitis may result in brain damage, hearing loss, or a learning disability in 10 to 20% of survivors	x	x	x
Mumps	Children aged 5–9 years most often affected	Deafness, meningitis (infection of the brain and spinal cord covering), painful swelling of the testicles or ovaries, and, rarely, death	x	x	x
Rubella	Children aged 4–9years most often affected	In women: arthritis, risks of miscarriage, congenital anomaly (deaf, blind, mentally retarded, or with heart or brain damage)	x	x	
Human papillomavirus (HPV)	Genital warts and HPV-related cancer: adolescents and young adults aged 16–25years	Precancerous cervical, vulvar, and vaginal lesions; cervical, vulvar, and vaginal cancer; genital warts		x	x

From Ref. (14).

**Table 2:** Type of benefits in economic evaluations of vaccinations: application to Hib vaccination

Perspective	Benefit categories	Definition	Hib-specific examples
Narrow	Health gains	Reduction in mortality through vaccination	Hundreds of thousands of children die each year from Hib disease
	Healthcare cost savings	Medical expenditure savings because vaccination prevents disease episodes	Hib disease leads to substantial healthcare costs
	Care-related productivity gains	Savings of parents’ productive time because vaccination avoids the need for missing work to take care of a sick child	Parental care of children suffering from Hib disease can contribute substantially to the overall cost of the disease
Broad	Outcome-related productivity gains	Increased productivity because vaccination improves cognition and physical strength, as well as school enrolment, attendance, and attainment	Hib meningitis is relatively common and leaves 15–35% of survivors with permanent disabilities, such as mental retardation or deafness, which can severely reduce cognition
	Behaviour-related productivity gains	Benefits accrue because vaccination improves child health and survival, and thereby changes household choices, such as fertility and consumption choices	Hundreds of thousands of children die each year from Hib disease
	Community externalities	Benefits accrue because vaccination improves outcomes among unvaccinated community members	Hib infections are treated with antibiotics, leading to the development of resistance. Hib vaccination can protect unvaccinated individuals through herd effects

From Ref. (12).

Other childhood diseases, such as mumps and varicella, can lead to serious complications, such as meningitis, and even death in adulthood. Childhood vaccination not only protects infants and young children from these debilitating diseases but also can provide protection to adults and the elderly through prevention of transmission from the younger individuals (12, 17).

## Medium- and long-term investment in adolescent and young adult vaccination programmes

Adolescents and young adults are at risk of many infectious diseases such as pertussis, meningococcal meningitis, as well as sexually transmissible diseases caused by pathogens such as hepatitis B or human papillomavirus (HPV). The consequences of an outbreak of measles in 2008–2011 in France highlighted that adolescents and young adults are particularly vulnerable to this disease (18). These infections can lead to short- and medium-term complications (e.g., severe cough from pertussis, brain damage from meningitis, and genital warts from HPV), as well as a long-term risk to develop HPV-related cancers and chronic liver disease from hepatitis B (Table 1). These complications all have substantial consequences on the social and economic activities of these populations. For example, it has been reported that when a member of a household had cervical cancer, behaviour such as daily food consumption and school attendance could change, and this could negatively impact both their educational attainment and earnings (6, 19, 20).

Thus, adolescent and young adult vaccination, through boosters or catch-up programmes, provides medium- and long-term return on public health investment by protecting this population from debilitating diseases that can impact their development prior to adulthood.

## Rapid health and productivity gain from adult vaccination programmes

Infectious diseases in adulthood can cause substantial disruption for family and professional activities with a cumulative economic impact when considered on a national scale. For example, the whole population is susceptible to influenza every year, although the extent of the risk and the consequences are dependent on the circulating strains. A professionally active person who has influenza-like illness will take, on average, 2 to 5 days of sick leave (21). When this is multiplied by the number of working individuals infected in different economic sectors, there is a substantial impact on the economic growth of a nation. Sick days are a burden to individuals as they can represent a considerable proportion of their earnings, but they also result in substantial losses for the firm, which reduces its profitability. In France, for example, it has been estimated that working adults had to stop working for a mean of 4 days for influenza (21, 22). In Norway, the mean number of working days lost for seasonal influenza annually was estimated to be 793,000, resulting in an estimated productivity loss of US$231 million (23). This is without taking into account the decline in economic activity due to mass working force absenteeism or the reduced productivity while at work (presenteeism), which may pose a substantial economic burden on firms due to the loss of productive output.

Another vaccine-preventable disease in adulthood with potential consequences on work productivity is herpes zoster (HZ), more commonly known as shingles. HZ arises from the reactivation of a varicella virus that remains dormant after a childhood episode of varicella. The reactivation can be caused by a number of factors, including a decline of the immune system with age. HZ can lead to debilitating pain-related complications, such as post-herpetic neuralgia (PHN). In a recent study, it was reported that two-thirds of working adults (aged 50–65) who had HZ stopped working and about 75% reported decreased effectiveness at work (i.e., presenteeism) during almost 2 days because of HZ or PHN (24). Maintaining a healthy and productive workforce is a key priority for public debt sustainability and economic growth. Vaccination programmes can also protect patients with chronic conditions, thereby leading to substantial economic benefits for those who are working, which contributes to the economy and government tax base. Patients with chronic diseases, such as diabetes or chronic heart disease, are at higher risk of infectious diseases (25). For instance, patients with diabetes, who represent about 10% of the population aged 25 years and over in Europe (26), are at higher risk of HZ than individuals without diabetes, as was observed in a recent US study in which diabetes was associated with 45 and 18% adjusted risks for HZ and PHN, respectively (27). A recent European study also demonstrated that people with underlying conditions accounted for the greatest share of total costs avoided due to influenza vaccination, as most of these people are productively employed (28). Thus, vaccination programmes, such as influenza or HZ, can offer a clear contribution to improving economic productivity and minimising workforce absenteeism by preventing infection and diseases, in particular in those with chronic conditions (10).

## Elderly vaccination programmes contribute to a more active and healthier ageing population in a context of unprecedented demographic challenge in Europe

The global population is ageing, and Europe is by far the oldest continent. After Japan, Germany and Italy have the 2nd and 3rd highest median ages in the world (29). It is estimated that the population aged ≥65 years will almost double from 87.5 million in 2010 to 152.6 million in 2060. The demographic old-age dependency ratio (i.e., the ratio of the number of people aged ≥65 years to those aged 15–64 years) is projected to increase from 26% in 2010 to 52% in 2060 in the EU (30). This means that, by 2060, the number of working-age people supporting each pensioner will drop by half, not only making state pensions harder to afford but also raising the question of who will take care of this ageing population from a health and social perspective. Thus, the changing demographic situation is a serious threat to the financial and social-economic sustainability of the EU member states. Ensuring the ageing society will remain independent for longer and continue contributing to the economy and to society is key. Therefore, rapid demographic ageing is not only a major societal challenge (in terms of public budgets, workforce, competitiveness, and quality of life) but also a major opportunity for new jobs and growth, also referred to as the *Silver Economy* (31).

We can understand why the elderly are at higher risk of infectious diseases due to their declining immune system (immunosenescence) and their higher risk of comorbidities (32). They also have a higher risk of severe outcomes from infections because these are also strongly associated with unhealthy lifestyle, dietary deficiency, and polymedication (33). Very common infectious diseases in the elderly, such as influenza, pneumococcal infections, and HZ, can lead to complications that may contribute to or accelerate their overall functional decline, sometimes leading even to death. When elderly patients are hospitalised, they often experience reduced mobility and activity levels, increasing their risk for functional decline and dependency. Keeping this population away from hospitals should contribute to keeping them active and healthier and, therefore, not only less dependent but also able to assist their wider family with, for instance, childcare (10, 34).

For example, the results from a study in the United States demonstrated the substantial effectiveness of influenza vaccination and its benefits to healthcare systems (35). Influenza vaccination for the 2010–2011 season prevented more than 75% of adult hospitalisation in those aged over 50 years (35). Vaccination, as a key element in a primary prevention strategy against influenza, pneumococcal diseases, and shingles, can thus play a crucial role in keeping the ageing population active and healthy.

## Macroeconomic impact of vaccination

Investment in vaccination thus offers a wide range of economic and intangible benefits that can potentiate gains for the individual and for society (10, 36). As such, vaccination not only is a healthcare sector issue but also has repercussions for wider economic planning and long-term economic progress. However, the conventional economic evaluations usually conducted for vaccination generally omit health-related productivity and macroeconomic improvements attributed to health status changes and, consequently, may not adequately reflect the broader economic benefits of vaccination. The scope of macroeconomic evaluation is quite different from the microeconomic assessments that are widely used in cost-effectiveness analyses for drug regimens. For vaccination, the focus is to capture in addition the rate of return of public health investment (37). For example, the results from one study demonstrated that the net benefit of 60 years of investment in polio vaccine was six-fold higher (approximately $180 billion) than the cost during that time (16). This estimated positive net benefit was essentially based on the savings in treatment costs without taking into account the intangible costs of productivity loss and death. Hence, additional methods should be considered to capture the full benefits of vaccination, such as assessment of vaccination's impact on absenteeism, presenteeism, or individuals’ lifetime earnings due to improved educational achievement.

**Fig. 2:**
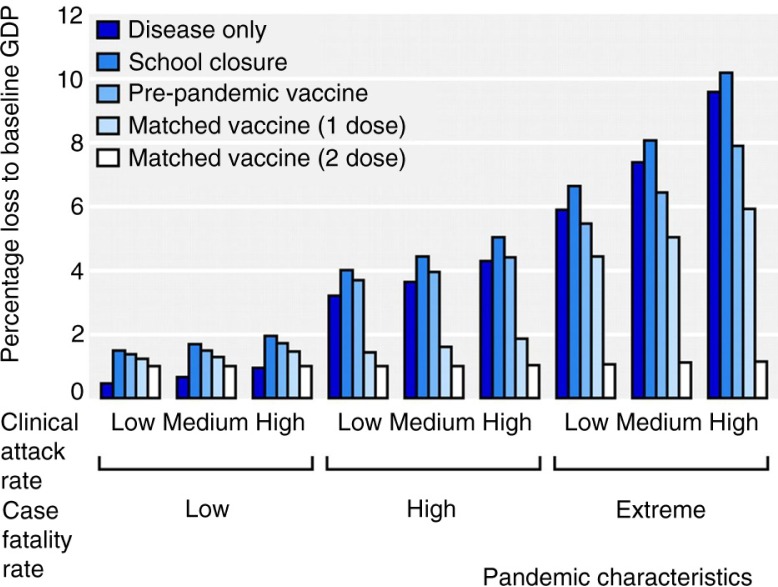
Effect of pandemic influenza on UK gross domestic product (GDP) according to various disease and mitigation scenarios (all strategies assumed to a 60% vaccine uptake) (38).

This approach has been used to evaluate the potential macroeconomic impact of pandemic influenza in the United Kingdom, including associated behavioural responses, school closure, and vaccination (38). The costs related to influenza alone ranged from between 0.5 and 1.0% of the GDP (between £8.4bn and £16.8bn) for high-fatality scenarios, and larger still for an extreme pandemic scenario. It was shown that vaccination with a pre-pandemic vaccine could save from 0.13 to 2.3% of the GDP (between £2.2bn and £38.6bn) over a single year (Fig. 2).

Other studies have estimated how lives saved could influence future government expenditure on social programmes such as health, education, and pensions, as well as influence future tax receipts. This is referred to as the ‘government perspective’ analysis and requires constructing a model that reflects the life course of average citizens, taking into consideration, for example, average schooling, employment, marriage, wages, and pension costs. Using this approach, a study conducted in Egypt concluded that investment costs in rotavirus vaccination for a cohort of infants would be entirely offset when the vaccinated cohort were 22 years old (39). Another study estimated the governmental return on investment for immunising adults aged 50 against diphtheria, tetanus, pertussis, seasonal influenza, pneumococcal diseases, and HZ in the Netherlands, by considering how investments in immunisation influence ongoing tax revenues to government (e.g., income tax, value-added tax, and social insurance contributions). Based on the investment costs of vaccinating adults aged 50, vaccination yielded a benefit–cost ratio of 4.09, suggesting a fourfold rate of return for the government (40).

Public investments in vaccination can therefore provide a significant public health benefit that translates into return on investment attributed to reduced government expenditure and increased tax revenues.

## Conclusions

Health is a key factor for the promotion of economic growth at the national, regional, and global levels. The vaccine industry and vaccination programmes targeted at populations of different ages can contribute substantially to economic growth by keeping people healthy throughout their lives, with continuous investment in research & development to protect populations against an increasing number of existing or new vaccine-preventable diseases. There is a clear need for a commitment to vaccination not only from health authorities but also from governments. In particular, the finance ministries and treasuries of different governments need to assess how best vaccines and vaccination can make an efficient contribution to their national economic growth, and thus to European growth. As such, macroeconomic analyses offer a critical evaluation tool but are rarely used. Greater impetus and investment in their use is needed to provide evidence to determine the full economic value of vaccination.

## References

[1] Mladovsky P, Srivastava D, Cylus J, Karanikolos M, Evetovits T, Thomson S Health policy responses to the financial crisis in Europe 2012.

[2] Loeppke R, Nicholson S, Taitel M, Sweeney M, Haufle V, Kessler RC (2008). The impact of an integrated population health enhancement and disease management program on employee health risk, health conditions, and productivity. Popul Health Manag.

[3] Suhrcke M, McKee M, Stuckler D, Sauto Arce R, Tsolova S, Mortensen J (2006). The contribution of health to the economy in the European Union. Public Health.

[4] Bloom DE, Canning D, Weston M (2005). The value of vaccination. World Econ.

[5] Reeves A, Basu S, McKee M, Meissner C, Stuckler D (2013). Does investment in the health sector promote or inhibit economic growth?. Global Health.

[6] Barnighausen T, Bloom DE, Cafiero-Fonseca ET, O'Brien JC (2014). Valuing vaccination. Proc Natl Acad Sci USA.

[7] Suhrcke M, Stuckler D, Suk JE, Desai M, Senek M, McKee M (2011). The impact of economic crises on communicable disease transmission and control: A systematic review of the evidence. PLoS One.

[8] Rémy V, Zöellner Y, Heckmann U (2015). Vaccination: The cornerstone of an efficient healthcare system. J Market Access Health Policy.

[9] Largeron N, Lévy P, Wasem J, Bresse X (2015). Role of vaccination in healthcare systems sustainability. J Market Access Health Policy.

[10] Postma M, Carroll S, Brandão A (2015). The societal impact of direct and indirect protection from lifespan vaccination. http://dx.doi.org/10.3402/jmahp.v3.26962.

[11] Deogaonkar R, Hutubessy R, van der Putten I, Evers S, Jit M (2012). Systematic review of studies evaluating the broader economic impact of vaccination in low and middle income countries. BMC Public Health.

[12] Barnighausen T, Bloom DE, Canning D, Friedman A, Levine OS, O'Brien J (2011). Rethinking the benefits and costs of childhood vaccination: The example of the Haemophilus influenzae type b vaccine. Vaccine.

[13] OECD (2012). Infant mortality. Health at a glance: Europe 2012.

[14] ECDC (2014). Health topics A–Z.

[15] ECDC Vaccine schedule 2014.

[16] ECDC Haemophilus influenzae: Factsheet for health professionals 2014.

[17] Thompson KM, Tebbens RJ (2006). Retrospective cost-effectiveness analyses for polio vaccination in the United States. Risk Anal.

[18] Antona D, Levy-Bruhl D, Baudon C, Freymuth F, Lamy M, Maine C (2013). Measles elimination efforts and 2008–2011 outbreak, France. Emerg Infect Dis.

[19] Arrossi S, Matos E, Zengarini N, Roth B, Sankaranayananan R, Parkin M (2007). The socio-economic impact of cervical cancer on patients and their families in Argentina, and its influence on radiotherapy compliance. Results from a cross-sectional study. Gynecol Oncol.

[20] Barnighausen T, Bloom DE, Cafiero ET, O'Brien JC (2012). Economic evaluation of vaccination: Capturing the full benefits, with an application to human papillomavirus. Clin Microbiol Infect.

[21] Keech M, Beardsworth P (2008). The impact of influenza on working days lost: A review of the literature. Pharmacoeconomics.

[22] Carrat F, Sahler C, Rogez S, Leruez-Ville M, Freymuth F, Le Gales C (2002). Influenza burden of illness: Estimates from a national prospective survey of household contacts in France. Arch Intern Med.

[23] Xue Y, Kristiansen IS, de Blasio BF (2010). Modeling the cost of influenza: The impact of missing costs of unreported complications and sick leave. BMC Public Health.

[24] Drolet M, Levin MJ, Schmader KE, Johnson R, Oxman MN, Patrick D (2012). Employment related productivity loss associated with herpes zoster and postherpetic neuralgia: A 6-month prospective study. Vaccine.

[25] Shah BR, Hux JE (2003). Quantifying the risk of infectious diseases for people with diabetes. Diabetes Care.

[26] World Health Organization (2010). The challenge of diabetes: Data and statistics.

[27] Suaya JA, Chen SY, Li Q, Burstin SJ, Levin MJ (2014). Incidence of herpes zoster and persistent post-zoster pain in adults with or without diabetes in the United States. Open Forum Infect Dis.

[28] Preaud E, Durand L, Macabeo B, Farkas N, Sloesen B, Palache A (2014). Annual public health and economic benefits of seasonal influenza vaccination: A European estimate. BMC Public Health.

[29] IndexMundi (2015). http://www.indexmundi.com.

[30] European Commission (2012). The 2012 ageing report: Economic and budgetary projections for the 27 EU Member States (2010–2060).

[31] Commission E (2015). Growing the European silver economy: Background paper.

[32] Heppner HJ, Cornel S, Peter W, Philipp B, Katrin S (2013). Infections in the elderly. Crit Care Clin.

[33] Aspinall R, Del Giudice G, Effros RB, Grubeck-Loebenstein B, Sambhara S (2007). Challenges for vaccination in the elderly. Immun Ageing.

[34] Kleinpell RM, Fletcher K, Jennings BM, Hughes RG (2008). Reducing functional decline in hospitalized elderly. Patient safety and quality: An evidence-based handbook for nurses.

[35] Talbot HK, Zhu Y, Chen Q, Williams JV, Thompson MG, Griffin MR (2013). Effectiveness of influenza vaccine for preventing laboratory-confirmed influenza hospitalizations in adults, 2011–2012 influenza season. Clin Infect Dis.

[36] Bonanni P, Picazo J, Rémy V (2015). The intangible benefits of vaccination – What is the true economic value of vaccination?. J Market Access Health Policy.

[37] Kotsopoulos N, Connolly MP (2014). Is the gap between micro- and macroeconomic assessments in health care well understood? The case of vaccination and potential remedies. J Market Access Health Policy.

[38] Smith RD, Keogh-Brown MR, Barnett T, Tait J (2009). The economy-wide impact of pandemic influenza on the UK: A computable general equilibrium modelling experiment. BMJ.

[39] Connolly MP, Topachevskyi O, Standaert B, Ortega O, Postma M (2012). The impact of rotavirus vaccination on discounted net tax revenue in Egypt: A government perspective analysis. Pharmacoeconomics.

[40] Kotsopoulos N, Connolly M Fiscal impact of adult vaccination in The Netherlands.

